# Visual motion thresholds mapped to midget and parasol ganglion cell topography in the human retina

**DOI:** 10.1038/s41598-025-16986-3

**Published:** 2025-09-01

**Authors:** Alexandra Hibble, Hannah Smithson, Paul Azzopardi

**Affiliations:** Department of Experimental Psychology, Life and Mind Building, University of Oxford, Worcester College, Walton St, South Parks Road, Oxford, OX1 3UD U.K.

**Keywords:** Neuroscience, Physiology, Psychology

## Abstract

Motion in visual images can be described in terms of changes in phases of Fourier components (phase cues), or displacements in the position of specific features (position cues) over time. Human observers are able to perceive motion using both cues, where perceived direction of motion is biased in favour of phase cues at higher spatial and temporal frequencies, and in favour of position cues at lower spatial and temporal frequencies. This suggests the existence of separable mechanisms for processing phase and position cues. We propose that these mechanisms receive separate inputs from the parasol (magnocellular) and midget (parvocellular) retinal ganglion cells. Using two-frame apparent motion Gabor stimuli that isolated phase and position cues, we measured displacement thresholds for motion direction discrimination across the visual field (from 0 to 15 degrees eccentricity) for 7 observers. Thresholds for positional displacements decreased significantly more steeply with eccentricity than those for phase displacements, mirroring precisely the decline with increasing eccentricity of the linear densities of the midget and parasol retinal ganglion cell populations respectively. These results suggest that the magnocellular and parvocellular visual pathways could constitute separable neural substrates for first-order (Fourier) and third-order (feature-tracking) motion perception.

## Introduction

### Psychophysical dissociations between phase and position cues for visual motion

**Fig. 1:**
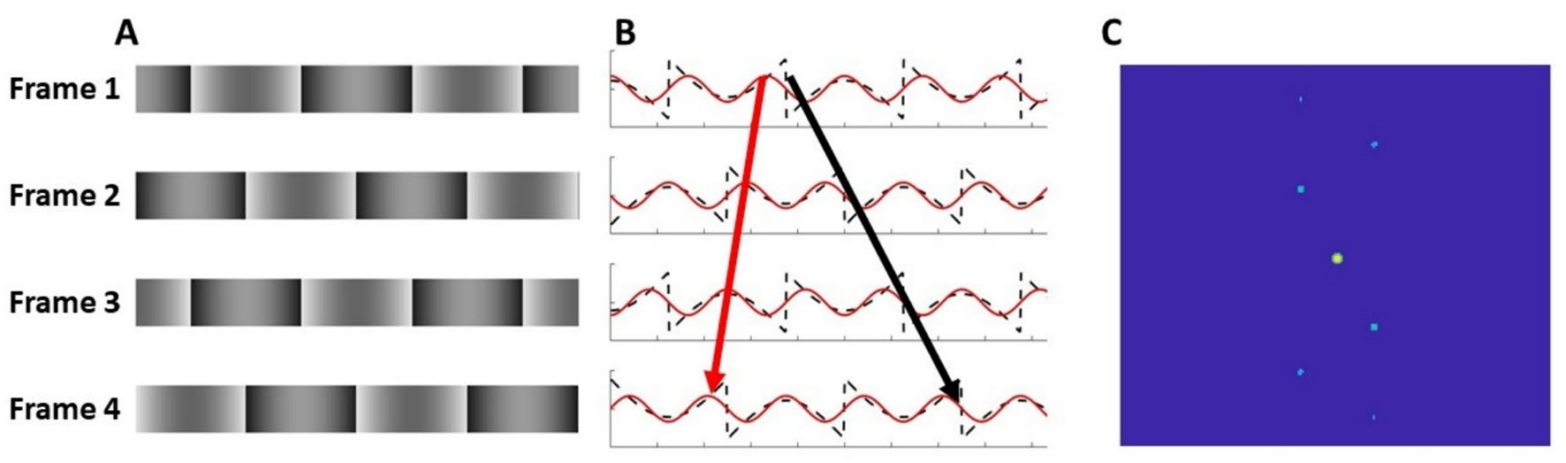
**A** Four frames of the Missing Fundamental Grating, with a 90° phase shift on each consecutive frame, with features displaced rightwards. **B**. Cross-section of each of the four frames in A. (dashed black line) with the third harmonic overlaid in red. The red arrow indicates the leftwards direction of the third harmonic, which is the lowest spatial frequency component of the stimulus, and the black arrow indicates the rightward direction of the features of the stimulus. **C**. Fourier analysis of the XT plane of a simulated 32 frame missing fundamental grating, where the pale blue peaks represent the frequency of the harmonic components. The distance of the peaks from the origin represents the spatial frequency of the input, with increasing distance representing higher spatial frequencies, the brightness of the peaks the amplitude (power), and the angle of the signals from this origin refer to the orientation of the frequencies in the XT input. The quadrants have been rearranged to display the lowest frequencies at the centre of the image, and progressively higher spatial frequencies towards the outside. This rearrangement means that the positive diagonal (bottom-left to top-right) corresponds to the leftward motion of the third harmonic, and not the rightward displacement of the features in the Missing Fundamental Grating.

**Fig. 2:**
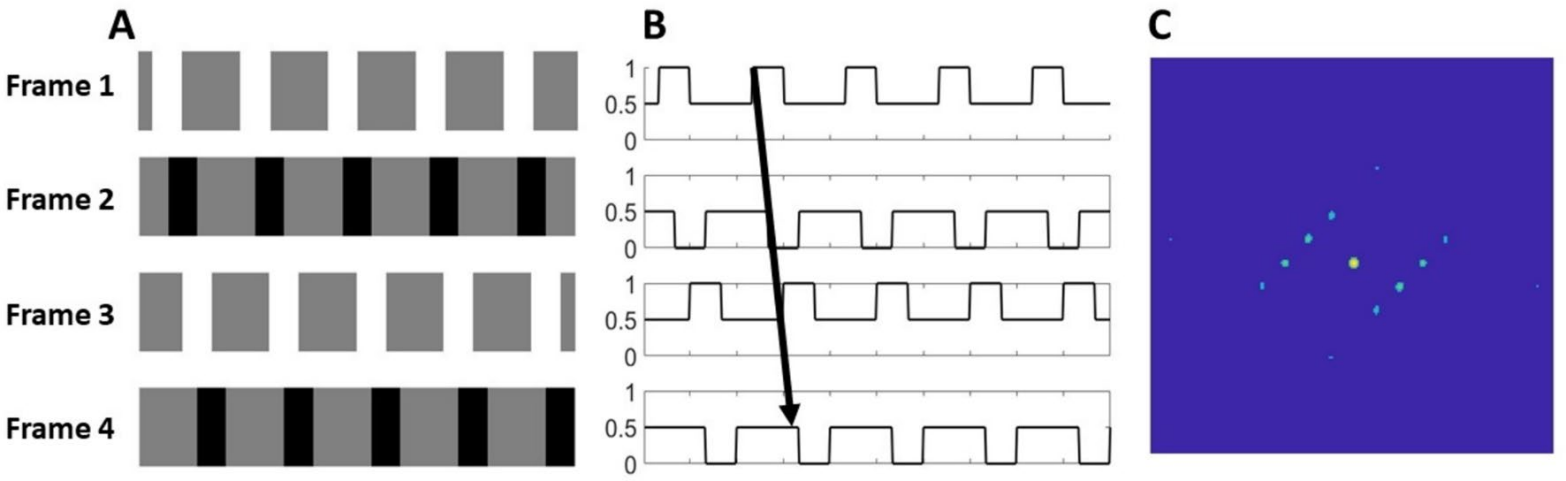
**A**. Four frames of a reverse-phi stimulus, where a square wave grating of duty cycle 0.25 has a simultaneous  45° phase shift and reversal of contrast on each frame. **B**. The cross-section of the reverse-phi grating in A. where the direction of feature displacement is indicated by the black arrow. **C**. Fourier analysis of the XT plane of a simulated 32 frame reverse-phi grating in A, where the pale blue peaks represent the frequency of the harmonic components. The positive diagonal (bottom-left to top-right) corresponds to the leftward motion of the frequency components in the image.

Motion in visual images can be specified in terms of the change in the spatial position of image features over time, or as the change in phases of the spatial frequency Fourier components of the image over time. Whilst, in theory, these should be mathematically equivalent, and should therefore signal motion in the same direction, apparent motion stimuli can be devised in which these two descriptions are dissociated, signalling motion in different directions. For example, a missing fundamental grating (Fig. 1, a square-wave grating with the fundamental spatial frequency component removed), displaced by the equivalent of one quarter of a cycle of the missing fundamental, produces two conflicting percepts: motion in the direction of the displacement, signalled by the change in position of features (*i.e.* high contrast edges, or regions of lightness), and motion in the opposite direction, signalled by aliasing of the phase of the next most salient (the third) harmonic^1,2^. Depending on the spatial and temporal parameters, observers may tend to perceive the latter by default, and only ‘see’ motion in the direction of displacement by effortful attention to features and tracking their displacement. Similar effects can be produced using 3f4f gratings (compound gratings consisting only of the 3^rd^ and 4^th^ harmonic components of a square-wave spectrum)^3^, and with reverse-phi stimuli (Fig. 2, in which a stimulus is displaced and its luminance contrast reversed simultaneously in successive frames)^4,5^, again due to aliasing in the frequency domain.

For brevity, we refer to a change in phase of the Fourier components as a ‘phase cue’ and a change in spatial position of image features as a ‘position cue’. The phase cue is analogous to first-order motion, which refers to motion produced by a drifting modulation of luminance, and the position cue is analogous to third-order motion, which refers to changes in the position of salient features, as defined by Lu & Sperling^6^. We use the terms ‘phase cue’ and ‘position cue’, as these describe the changes to the apparent motion stimuli across frames, without making assumptions about whether these cues are available to systems performing a first-order or third-order analysis. Normally, stimuli may contain multiple phase and position cues, signalling motion in many congruent and conflicting directions simultaneously.

Either cue in isolation is sufficient for human observers to perceive visual motion. For phase, this has been shown using stimuli that contain coherent motion in low-contrast, pedestalled, sine waves where the contrast-defined features oscillate back and forth (obscuring coherent direction for changes feature position), but the change in phase of the sine wave signals motion in a consistent and coherent direction^7^. Conversely, the phase cue can be silenced in stimuli where frames with different visual features are interleaved so that displacement has to be mapped across features with different components, and the stimulus therefore has no coherent Fourier components between frames, as demonstrated with MF1F gratings, in which successive frames alternate between a missing fundamental grating and a sinewave corresponding to the fundamental^8^, and with sets of interleaved gratings reliant on attention^9^.

When cues are combined in such a way as to signal motion simultaneously in opposing directions, such as with reverse-phi stimuli, observers consistently report motion in the phase cue direction at high spatial and temporal frequencies, and in low contrast stimuli, and in the position cue direction at lower spatial and temporal frequencies (reverse-phi^4,10–12^^,13^; spatial frequency filtered random dot kinetograms^14^). The relationship with temporal frequency has been replicated in other stimulus constructions, with a bias in reporting motion in the phase cue direction at high temporal frequencies (Missing Fundamental Gratings^15,16^, drifting orthogonal Gabor patches^17,18^). Thus, human observers are sensitive to both phase and position cues, with distinct spatiotemporal sensitivity profiles for each. This might suggest the existence of two separate motion processing systems.

Further evidence suggesting that phase and position cues may, at least initially, be processed by separate mechanisms comes from studies that demonstrate that phase cues are processed monocularly, but that motion signalled by position cues can be successfully perceived when stimuli are presented dichoptically. This has been shown using pedestaled luminance- and contrast-modulated gratings^9^, reverse-phi stimuli^19^ and Missing Fundamental Gratings^16^.

The qualitative changes in motion perception across the retina, where motion is biased towards the position cue when viewed foveally, and in the phase cue direction when viewed peripherally, are also suggestive of psychophysical dissociation between phase and position cues. This has been observed in reverse-phi stimuli^5,10,11,19,20^, and also with drifting Gabor stimuli with orthogonal motion of the carrier and aperture, where perceived motion is biased towards the displacement of the aperture when viewed foveally, and biased in the direction of the phase changes of the carrier when viewed peripherally (Shapiro et al*.*, 2010^18^). The change in reported motion direction for these stimuli between the fovea and periphery could result from a change in the relative sensitivity of mechanisms processing phase and position cues across the retina.

### Neural correlates of phase and position cues for visual motion

The visual system relies on two major pathways to convey information from the retina to the visual cortex. The parvocellular pathway originates from the midget ganglion cells of the retina, projecting through the parvocellular laminae of the dorsal lateral geniculate nucleus (dLGN) of the thalamus to layer 4Cβ of the primary visual cortex, and it accounts for *c.* 80% of retinal ganglion cells and dLGN relay neurons; the magnocellular pathway originates from the parasol ganglion cells of the retina, projecting through the magnocellular laminae of the dLGN to layer 4Cα of the primary visual cortex, accounting for *c.* 10% of retinal ganglion cells and dLGN relay neurons. Functionally, there are important distinctions between these two pathways. Neurons in the parvocellular pathway have relatively small receptive fields, respond selectively to the wavelength of light, and have sustained responses, whereas neurons in the magnocellular pathway have larger receptive fields, are broadband (*i.e.* insensitive to the wavelength of light), have transient responses, and are more sensitive to low contrast. Although the two pathways are anatomically separate as far as a primary visual cortex, and to some extent through extrastriate cortex, there is a certain amount of overlap between them in the range of spatial and temporal sensitivities with midget system tuned to higher spatial frequencies and lower temporal frequencies, and the parasol system favouring lower spatial frequencies and higher temporal frequencies.

The role of the parasol RGCs in motion perception is well reported, where lesioning the magnocellular layers of the LGN (where the majority of parasol RGCs project to^21^) in macaques dramatically impairs temporal acuity, measured by critical-flicker-fusion thresholds^22^, and discrimination of stimuli at higher temporal frequencies^23^. Similarly, the receptive fields of magnocellular cells in the dLGN are tuned to higher temporal frequencies than the parvocellular cells^24–26^, which receive input from the midget ganglion cells^21^. The role of the midget ganglion cells is more often discussed in terms of their role in shape and position perception^27^, and not in relation to motion perception, even for position cues. Visual form processing therefore tends to be ascribed to the midget system and motion to the parasol system, although this is likely to be an oversimplification (see^22,27^ and^28^ for reviews of the very extensive literature). It is possible that motion perception from position cues is ‘piggy-backing’ onto this midget system, requiring the discrimination of position to detect the displacement of features.

Psychophysical thresholds correlate with parvocellular substrates for static stimuli, demonstrating the critical role of the midget ganglion cells in the discrimination of both pattern^13^, and chromatic stimuli^29^. The density of ganglion cells varies dramatically across the retina, with estimates ranging from as high as ~ 32,000–38,000 cells/mm^2^ at the fovea, decreasing to as low as 500 cells/mm^2^ in the peripheral retina corresponding to eccentricities exceeding 35° from the fovea^30^. Furthermore, the midget and parasol ganglion cell populations have distinctly different topographies, with the density of midget cells declining far more steeply between fovea and periphery than the densities of parasol cells (see Fig. 5)^30^. The density of midget RGC can be considered as the “fundamental limit on the spatial resolution of human vision”^13^, as they are the initial sampling units for the magnocellular pathway implicated in processing of shape and colour. Here, we extend this approach to the parasol ganglion cells, whereby the relatively shallower decline of parasol ganglion cell density with eccentricity^29^ might explain the relatively shallower decline in motion thresholds with eccentricity when compared to Vernier thresholds and minimal angle of resolution^31^, or Vernier thresholds and grating acuity^32^: data from Westheimer, 1972 and Westheimer, 1989).

### Aims & hypotheses

We consider whether sensitivity to phase and position cues can be dissociated psychophysically, and whether motion discrimination performance based on the different cues correlates with differences in the topography of magnocellular and parvocellular substrates.

We measured displacement thresholds for direction discrimination at four retinal eccentricities in the temporal visual field (nasal retina) between the fovea and 15° from seven observers using two-frame, apparent motion Gabor stimuli presented at seven spatial frequencies with a temporal, 2-alternative forced choice (2AFC) procedure. The phase cue was generated by shifting the phase of the sinusoidal carrier grating by a quarter cycle (90°) behind a static window between frames, while the position cue was generated by displacing the Gaussian window by an equivalent distance while eliminating the phase cue by rotating the carrier 90° between frames. At each eccentricity, an observer’s displacement threshold was taken as one-quarter of a cycle of the highest spatial frequency at which the observer could correctly report motion direction in 75% of trials. Then, we compared the slopes of these psychophysical thresholds as a function of eccentricity with published models of the topography of parasol and midget ganglion cells in the human retina, hypothesising that these would correspond to the displacement thresholds for phase and position cues respectively.

## Results

### Thresholds for motion-direction discrimination

**Fig. 3:**
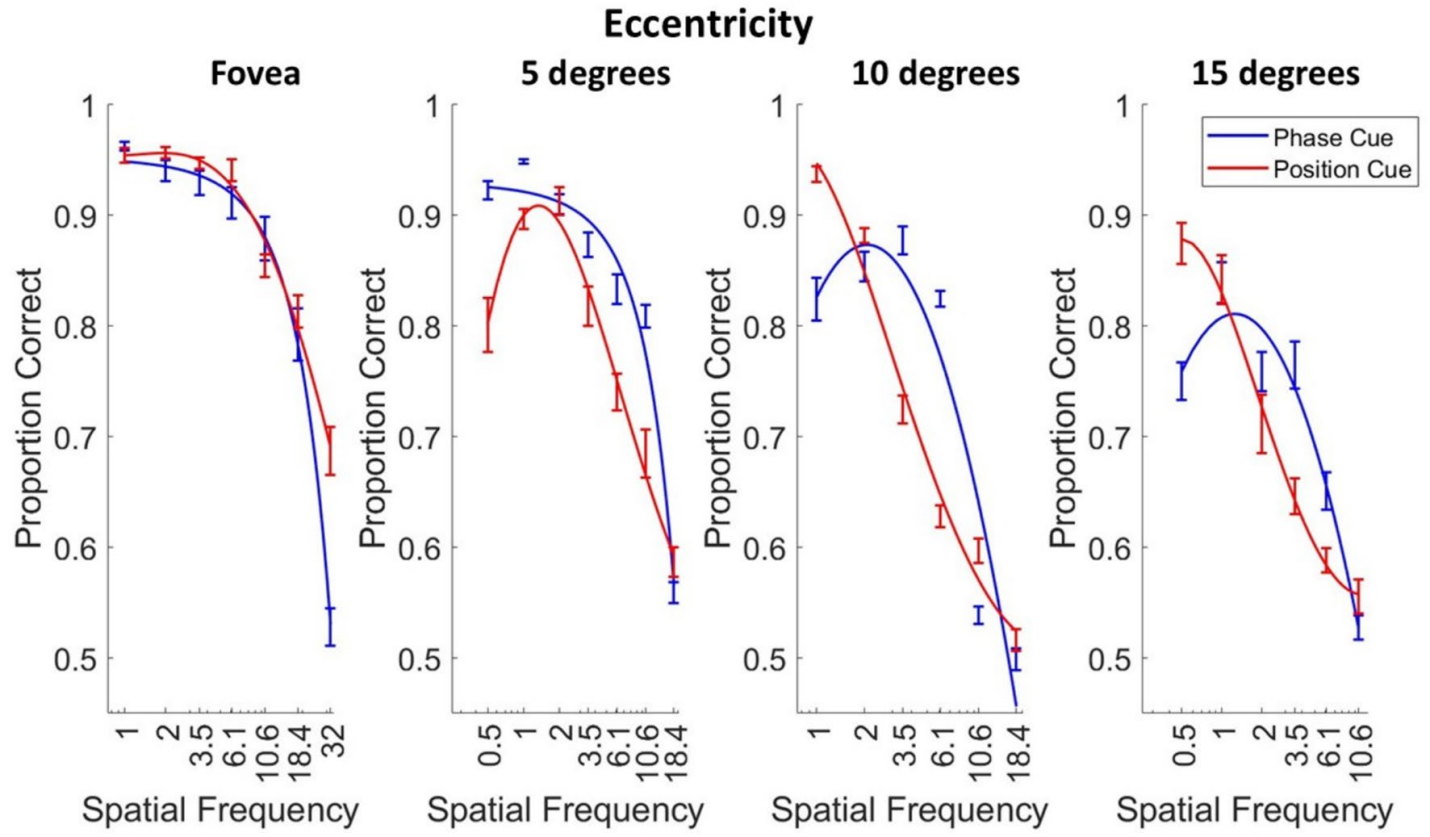
Performance of seven observers in a two-alternative, forced-choice motion direction discrimination task across a range of spatial frequencies, at 4 retinal locations along the horizontal meridian of the nasal retina: fovea, 5°, 10° and 15° eccentricity. Observers reported the interval in which the Gabor stimulus moved upwards, where the motion of the Gabor was either cued by a change in the phase of the carrier (phase cue), or the displacement of the Gaussian envelope with a simultaneous 90° rotation of the carrier, to eliminate relative phase cues (position cue). Mean proportion correct is plotted as a function of spatial frequency separately for the two cues (phase in blue, and position in red), for each of the 4 retinal locations. The bars represent the standard errors of the mean. Three-parameter quadratic functions were fitted separately to the proportion correct scores in the phase and position conditions at each of the four eccentricities.

First, we measured the ability of seven observers to discriminate the direction of motion depicted in two-frame kinematograms as a function of spatial frequency, retinal eccentricity, and whether the motion was cued by a change in phase or position. The stimuli were Gabor patches in which the phase cue was generated by shifting the phase of the sinusoidal carrier grating by a quarter of a cycle (90°) behind a static, Gaussian-weighted window, whereas the position cue was generated by displacing the Gaussian-weighted window by an equivalent distance while eliminating the phase cue by rotating the carrier by 90° between frames. The tests were conducted at four eccentricities (0°, 5°, 10°, and 15°) along the horizontal meridian of the nasal retina, and up to seven spatial frequencies (ranging from 0.5 to 32 cycle per degree, but with a different set of spatial frequencies according to eccentricity, described in the Methods section). The four eccentricities used were deemed sufficient on the basis of pre-existing literature^33–35^ to differentiate threshold gradients as function of eccentricity for the purpose of testing our hypothesis. Observers’ ability to discriminate direction of motion was measured by means of temporal, two-alternative forced-choice (2AFC) tests in which, on each trial, a pair of two-frame kinematograms, one depicting upwards motion, and one depicting downward motion, were presented in randomised order, and the observer was asked to indicate the interval in which upward motion was depicted. These data are summarised in Fig. 3.

### Analysis of high spatial frequency cutoffs

**Fig. 4:**
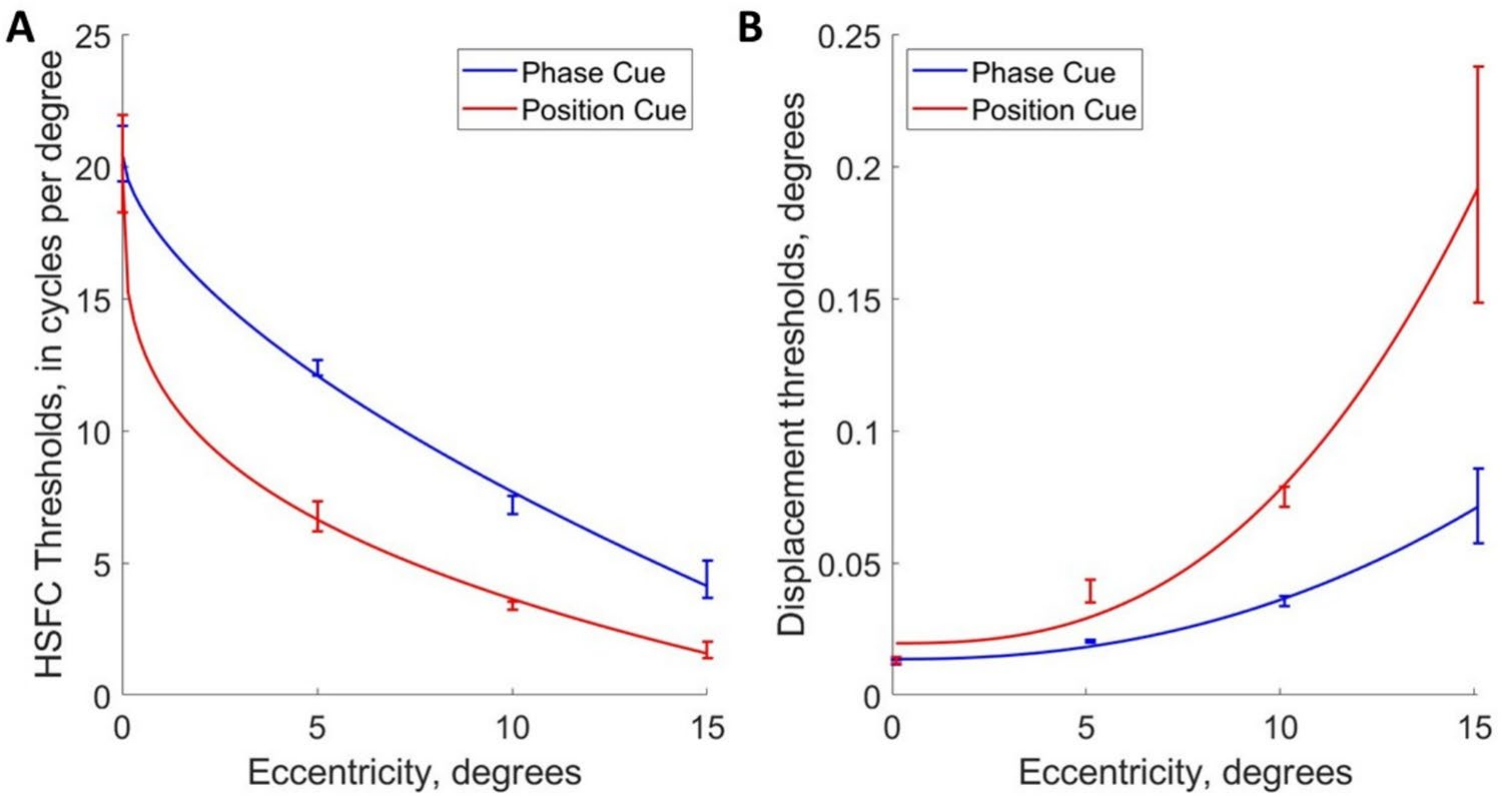
The three-parameter quadratic functions summarised in Fig. 3 were plotted individually for each observer, and the spatial frequency corresponding to 75% correct direction discrimination (the high frequency cutoff) was obtained by interpolation. **A**. Two-parameter power functions were fitted to the mean high spatial frequency cutoffs (HSFC) across the 7 observers for motion-direction discrimination in phase-cued (blue) and position-cued (red) apparent motion, as a function of retinal eccentricity along the horizontal meridian of the nasal retina. The bars represent the standard error of the mean. **B**. These high spatial frequency cutoffs were transformed into displacement thresholds (the reciprocal of a quarter of the interpolated wavelength), to visualise the minimum displacement threshold required for observers to achieve 75% correct direction discrimination, as a function of retinal eccentricity. These data were fitted by a two-parameter power function separately for the phase-cued (blue) and position-cued (red) apparent motion conditions, where the bars represent the standard errors of the mean.

Next, we interpolated, at each eccentricity, for each observer, the highest spatial frequency at which the observer correctly reported motion direction depicted on 75% of trials. These data are summarised in Fig. 4A, which shows that these high spatial frequency cutoffs (HSFC) decrease more steeply with eccentricity for position-cued direction discrimination than for phase-cued direction discrimination.

The changes in high spatial frequency cutoffs with eccentricity approximate power functions, which, when plotted on log–log axes, map onto straight lines where the slope corresponds to the exponent of the power function. After applying log–log transformations to the data, linear mixed-effects models were used to test whether the decrease in observers’ high spatial frequency cutoffs with eccentricity was significantly different between the thresholds for phase- and position cued motion discrimination. The full model included fixed effects for log eccentricity, motion cue, and their interaction, with a random intercept for observer, and the reduced model excluded motion cue as a fixed effect.

A likelihood ratio test comparing the full model (including motion cues) to the reduced model (collapsed across motion cues) showed that including motion cues significantly improved the model fit, χ^2^(2) = 25.711, *p* < 0.001. The full model explained a greater portion of the variance (marginal R^2^ = 0.745) compared to the null model (marginal R^2^ = 0.609), a large effect size (Cohen’s f^2^ = 0.587).

There was a significant main effect of motion cue (*b* = −0.178, *SE* = 0.062, *t*(46) = −2.902, *p* = 0.006), a significant main effect of eccentricity (*b* = −0.264, *SE* = 0.044, *t*(46) = −6.012, *p* < 0.001), and a significant interaction between motion cues and eccentricity (*b* = −0.169, *SE* = 0.062, *t*(46) = −2.717, *p* = 0.009). This analysis shows that the phase-cued and position-cued high spatial frequency cutoffs change at significantly different rates with increasing retinal eccentricity.

### Analysis of displacement thresholds

High spatial frequency cutoffs can also be plotted as displacement thresholds (Fig. 4B), calculated by taking the reciprocal of a quarter of the interpolated wavelength.

An established way of comparing displacement threshold gradients (Fig. 4B) as a function of eccentricity is to calculate the ‘E2 value’, which refers to the eccentricity at which a stimulus must be doubled in size to achieve the same as foveal sensitivity^36^. E2 values for phase-cued and position-cued apparent motion were obtained for each observer by fitting power functions to their displacement thresholds (r^2^ > 0.95 for every combination of cue and observer) and then interpolating the eccentricity corresponding to a doubling of the foveal displacement value. E2 values for phase-cued direction discrimination (mean = 6.93°, SE = 0.23) were significantly higher than for position-cued direction discrimination (mean = 2.40 ^o^, SE = 0.22) (paired-sample t-test: t = 4.539, df = 6, *p* = 0.004, Cohen’s *d* = 1.716).

### Correlation between high spatial frequency cutoffs and ganglion cell densities

**Fig. 5:**
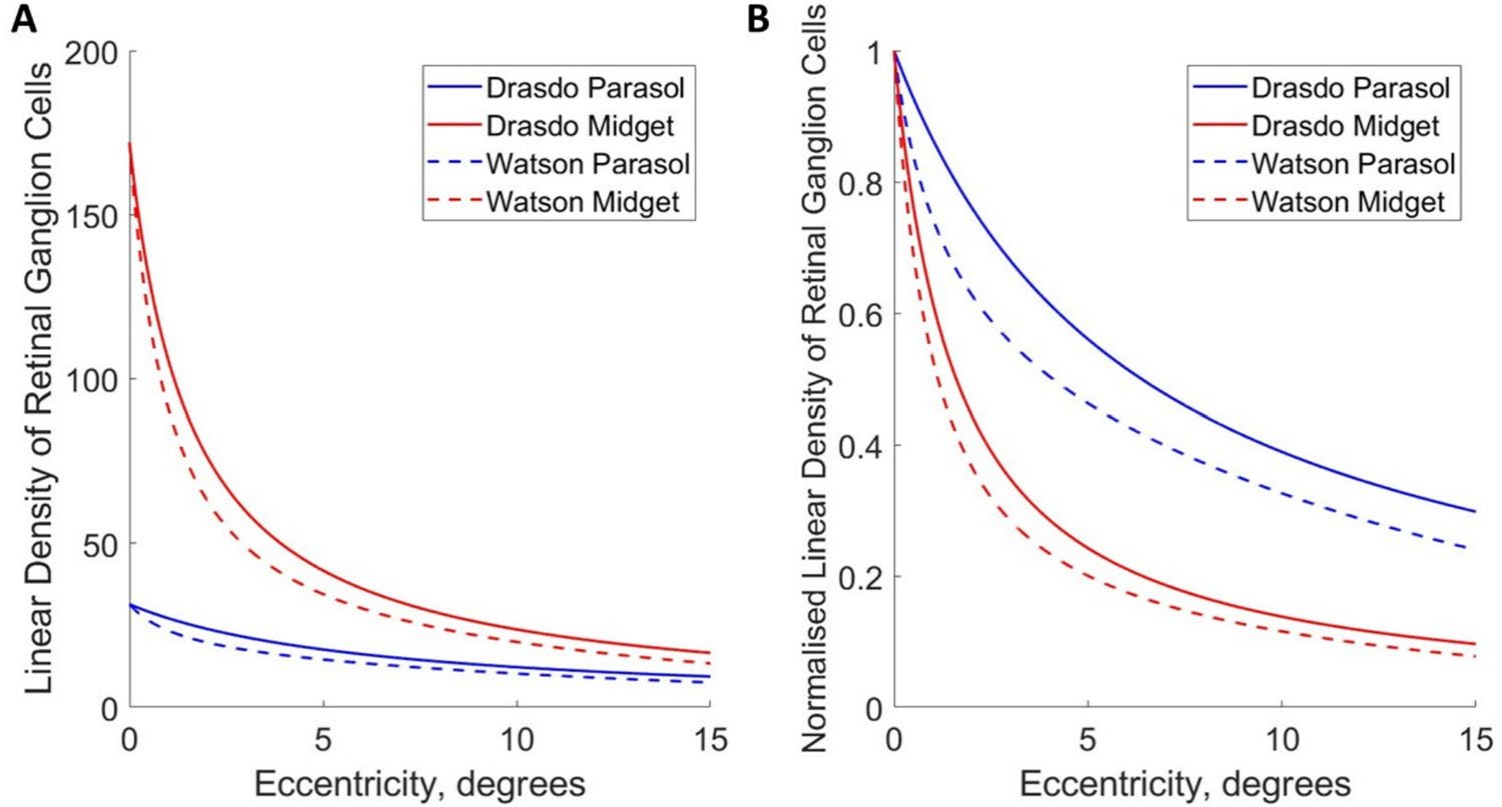
**A** Models of ganglion cell density across horizontal meridian along the horizontal meridian of the nasal retina, derived from Watson (2014)^13^, and Drasdo (1989)^37^, expressed as linear density (ganglion cells per degree). The derivation of these models is discussed in the methods section. **B**. The same models normalised with respect to their values at the fovea, demonstrating the relatively steeper decline in the density of midget ganglion cells (in red) with eccentricity than parasol ganglion cells (in blue).

Models of parasol and midget ganglion cell density between 0° and 15° degrees of eccentricity along the nasal retina were derived from Watson (2014)^13^, and Drasdo (1989)^37^ (see Fig. 5, and the Methods section for details on their construction).

**Fig. 6:**
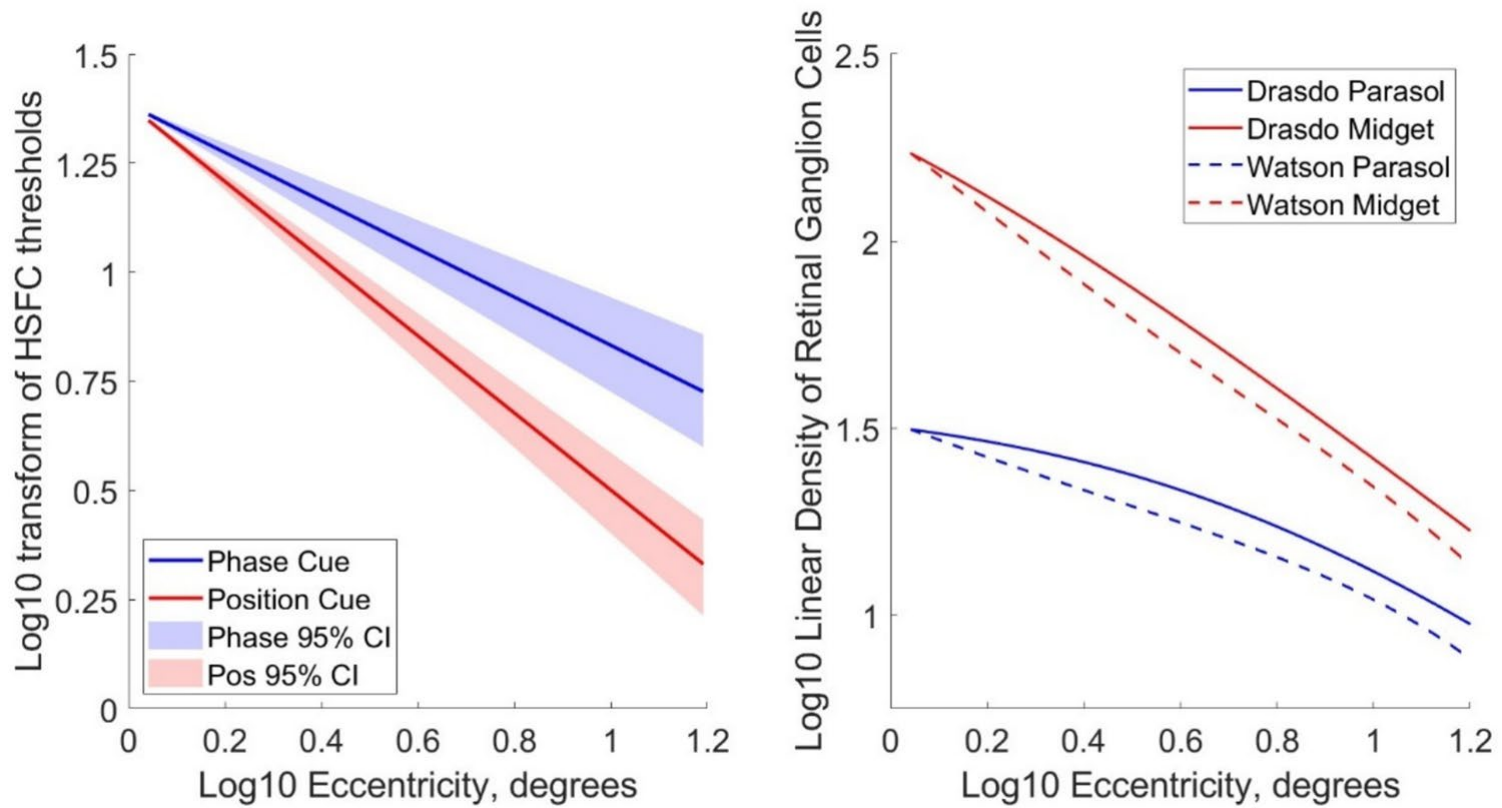
**A** The high spatial frequency cutoffs (HSFC) for phase and position cues presented in Fig. 4A were plotted on log–log axes and fitted with linear regressions, where the shaded areas represent the 95% confidence intervals across the 7 observers. **B**. Linear densities of retinal ganglion cells (ganglion cells per degree), derived from Watson (2014)^13^ and Drasdo (1989)^37^ presented in Fig. 5A are plotted on log–log axes. These can be approximated by power functions obtained by fitting linear regressions to the log–log transformed data.

Both ganglion cell density distribution models and the observers’ high spatial frequency cutoff data can be approximated by power functions (see Fig. 6). Ganglion cell density is expressed as linear density (ganglion cells per degree, calculated as the square root of the planar density), as is appropriate for the 1D spatial frequency stimuli we used in this psychophysical experiment.

By applying log transformations to these data and eccentricity, linear regressions can be fitted to these data, allowing us to compare the relative slopes of the regressions.

The high spatial frequency cutoff data were log10-transformed and modelled as a function of the log10-transformed eccentricity values, offset by 1.1 degrees to avoid undefined values at or near zero eccentricity. These log-transformed thresholds for phase and position cue conditions were fitted with linear models separately for each observer, producing values for the slope and intercept for each observer.

**Fig. 7:**
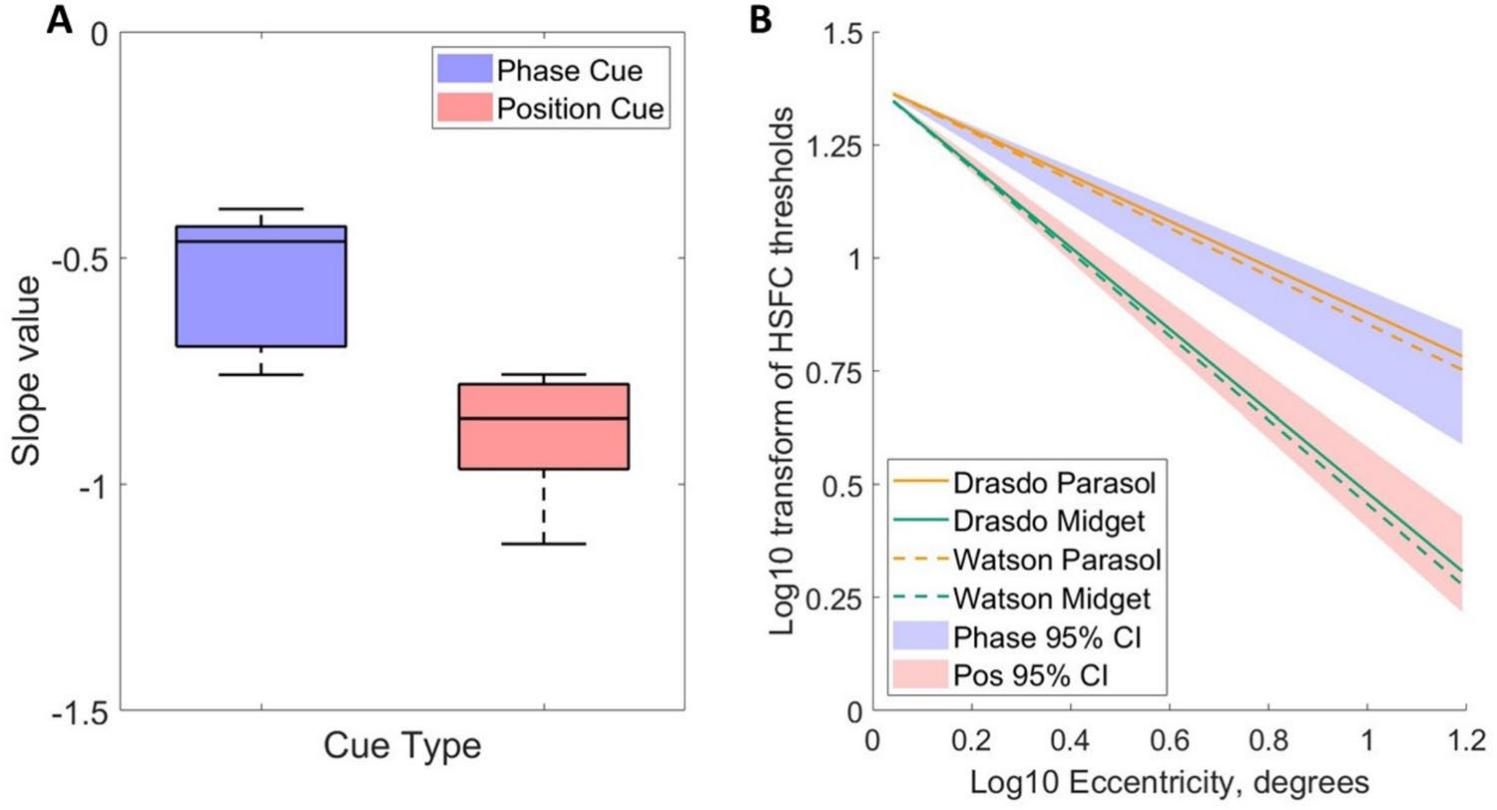
**A** Boxplots displaying the slope values for the linear regressions fitted to observers’ log–log transformed high spatial frequency cutoff values plotted against eccentricity, separately for the phase (in blue) and position (in red) cues. **B**. This graph compares the linear regressions fitted to observers’ high spatial frequency cutoffs for phase (blue) and position (red) cues (shown in Fig. 6A), with the linear regressions fitted to the linear density models of midget (in green) and parasol (in orange) RGC density as ganglion cells per linear degree for the Drasdo (1989)^37^ and Watson (2014)^13^ models. To demonstrate that the regressions have almost identical slopes, the regressions based on cell density were aligned vertically with the psychophysical data by plotting them with the averaged intercept of the psychophysical regressions.

Slope values were significantly greater in the position cue condition (mean = −0.884, SD = 0.136), than the phase cue condition (mean = −0.552, SD = 0.154), as measured by a paired samples t-test (t(6) = 6.425, *p* < 0.001, Cohen’s *d* = 2.428), (see Fig. 7A). Intercept values were not significantly different between the phase and position cue conditions (*p* = 0.963; position cue condition, mean = 1.383, SD = 0.114; phase cue condition, mean = 1.386, SD = 0.095).

Similarly, the ganglion cell density distribution models of Watson (2014) ^13^ and Drasdo (1989) can be approximated by power functions obtained by fitting linear regressions to the log–log transformed data (Fig. 6B), allowing us to compare the relative slopes of the regressions for cell density data and psychophysical data. The slopes and intercepts for the regressions fitted to the log transformed parasol and midget cell density distributions (expressed in ganglion cells per degree) were similar for the Watson (2014) ^13^ and Drasdo (1989)^37^ models:

Watson (2014) ^13^ midget RGC model: ${\text{log}}_{10}\left({\text{density}}\right) = -0.929\cdot {\text{eccentricity}}+2.264$

^Drasdo (1989)37^ midget RGC model: ${\text{log}}_{10}\left({\text{density}}\right)=-0.903\cdot {\text{eccentricity}}+2.320$

Watson (2014)^13^ parasol RGC model: ${\text{log}}_{10}\left({\text{density}}\right)=-0.531\cdot {\text{eccentricity}}+1.559$

^Drasdo (1989)37^ parasol RGC model: ${\text{log}}_{10}\left({\text{density}}\right)=-0.505\cdot {\text{eccentricity}}+1.61$

The slopes of the linear regressions fitted to the log–log transforms of the ganglion cell densities as a function of eccentricity were compared with the slopes of observers’ high spatial frequency cutoffs as a function of eccentricity for phase- and position-cued motion. The slopes of the models of parasol retinal ganglion cell density derived from Watson (2014) ^13^,slope = −0.531), and from Drasdo (1989)^37^; slope = −0.505), fall clearly within the 95% confidence intervals of the slopes of the observers’ high spatial frequency cutoffs for the phase cue (mean slope = −0.552, 95% CI [–0.696, –0.431]). The slopes of the models of midget ganglion cells density (Watson (2014) ^13^: slope = −0.929; Drasdo (1989)^37^: slope = −0.903) fall clearly within the 95% confidence intervals of the slopes of the observers’ high spatial frequency cutoffs for the position cue (mean slope = −0.884, 95% CI [–0.966, –0.779]). The close correspondence between the psychophysical performance and the anatomical data is demonstrated in Fig. 7B, which depicts the linear regressions describing the ganglion cells densities superimposed on the linear regressions describing the high spatial frequency cutoffs, aligned by using the intercept of the psychophysical regressions for both the psychophysical and the anatomical data.

We can also compare the relative decline in density of the midget and parasol cells with eccentricity, and the relative decline in observers’ sensitivity to phase and position cues. The density of midget retinal ganglion cells with eccentricity declines more rapidly than the density of parasol ganglion cells (see Fig. 5). If the relative decline for the position-cue HSFC compared to the phase-cue HSFC for each observer is similar to the ratio of decline between the midget and parasol retinal ganglion cells, this would offer further support for arguing that the midget retinal ganglion cells subserve position-cue HSFC, and the parasol retinal ganglion cells subserve phase-cue HSFC. The mean ratio for observers between the slope values for the position and phase cue conditions was ratio of 1.675 [95% CIs: 1.346, 2.003]. The ratios of slopes for ganglion cell densities fall comfortably within these 95% confidence intervals (ratio of midget to parasol cell slopes for Watson (2014)^13^=1.750; Drasdo (1989)^37^= 1.787), suggesting that the relative decline of observers’ sensitivity to position cues relative to their sensitivity to phase cues, follows the same relationship as the ratio of the decline in the relative number of parasol to midget ganglion cells with eccentricity.

## Discussion

We hypothesised that the steeper decline in sensitivity to position cues, compared to phase cues, with increasing retinal eccentricity could suggest that phase and position cues are processed (at least initially) by separable neural substrates, namely the parasol and midget retinal ganglion cells (RGC).

Consistent with this hypothesis, we found that observers’ high spatial frequency cutoffs declined significantly more steeply in the position cue condition than in the phase cue condition. This replicates previous findings showing a peripheral bias toward phase cues over position cues in motion processing, as shown in conflicting cue stimuli (reverse-phi stimuli^11^:,drifting orthogonal Gabor: Shapiro et al*.*, 2010), and could explain the phenomenon of preserved motion perception in stimuli where the position of the start and end locations of the motion stimulus could not be perceived (Exner, 1875^33^).

We compared the decline in observers’ high spatial frequency cutoffs with eccentricity for the phase and position cues, with the rates of decline in parasol and midget retinal ganglion cell density with eccentricity, derived from Watson (2014)^13^, and Drasdo (1989)^37^. The decline in parasol and midget RGC density with eccentricity was approximated by a linear regression fitted to log10 transformed linear ganglion cell density per degree and log10 retinal eccentricity in degrees. The slopes of these regressions can be compared to the slopes of the linear regressions modelling observers’ decline in HSFC with eccentricity for the phase and position cue conditions. The slope, modelling the decline in density, for parasol RGC density for both Watson (2014)^13^ and Drasdo (1989)^37^ fell within the 95% confidence intervals for the slopes of observers HSFC in the phase cue condition. Similarly, the slope for midget RGC density for both Watson (2014)^13^ and Drasdo (1989)^37^ fell within the 95% confidence intervals for the slopes of observers HSFC in the position cue condition.

This is suggestive of a relationship between the change in density of parasol RGCs with eccentricity and sensitivity to phase cues for motion perception, and likewise, of a relationship between the change in density of midget RGCs with eccentricity and sensitivity to position cues for motion perception. This could be interpreted as the parasol and midget RGCs producing separable sampling resolution limits for the processing of phase and position cues.

Another method to assess the relationship between the retinal ganglion cell topography and psychophysical thresholds is to compare whether the relative change with eccentricity for the midget and parasol RGC distributions, is similar to the relative change with eccentricity in the high spatial frequency cutoffs measured in the position and phase cue conditions. We assessed this by taking the ratio of the slopes of the midget and parasol cell density models with eccentricity for both the data interpolated from Drasdo (1989)^37^ and Watson (2014)^13^ and comparing these slope ratio values to the ratios of the observers’ high spatial frequency cutoffs with eccentricity in the position and phase cue conditions. The slope ratio values of the cell density models fell within the 95% confidence intervals of the observers’ slope ratio models, suggesting that the changing sensitivity to phase and position cues across the retina follows the same rate of change as the changing ratio of midget to parasol cells.

Taken together, both these results are suggestive that the parasol and midget ganglion cells may be separably processing the phase and position cues for visual motion.

A possible alternative explanation for these results is the different spatial scale of the phase and position cues. Although both cues are presented within a Gabor stimulus, the phase cue (displacement of the carrier) could be perceived at the scale of one cycle of the carrier whilst the position cue (displacement of the Gaussian envelope) is a ‘centre of gravity task’, where, due to the rotation of the carrier between frames, the change in envelope position requires integrating across the whole stimulus, where the Gabor contains three visible cycles of the carrier. Along with the decline of spatial resolution in the periphery^13^, position uncertainty increases with eccentricity^38–40^. Both these factors may cause a relatively greater decline in sensitivity to position cues than phase cues due to this difference in task demands, rather than reflecting a difference in the neural substrates subserving the processing of phase and position cues.

Support for our findings can be found from previous studies that compare threshold gradients across the retina, which, despite using different stimuli, have found similar gradients in the decline of spatial frequency thresholds (or, if using the displacement thresholds, then the increase in displacement thresholds with eccentricity) to the gradients presented in this paper when testing sensitivity to motion and position. We can compare between these gradients for different stimuli using Levi’s E2^36^, which refers to the eccentricity at which a stimulus must be doubled in size to achieve the same sensitivity as foveal sensitivity. The E2 values for observers in the phase cue condition (mean = 6.93°, SE = 0.23) were similar to those reported in tasks using unreferenced motion produced from gratings^32^: 5.8^o^ and 10.5^o^) and dots^41^,6.3, 7.9, 8.6 and 11.1^o^). The E2 values for observers in the position cue condition (mean = 2.40^o^, SE = 0.22) were similar to those reported for acuity for static gratings (reported in^42^. Grating acuity^43^: 2.6^o^, 2.7^o^^;[44^ 2.7^o^^;[36^, 2.6^o^-3.0^o^^;[31^, ~ 2.5^o^^45^,3.0^o^). The marked similarity in E2 values between our data and these published values, despite different stimulus designs requiring integration over different sized spatial areas, is also suggestive that the changes in sensitivity with eccentricity reflect the presence of different mechanisms for processing phase and position cues rather than a difference in spatial scale between the phase and position cues.

An alternative source of evidence that supports an interpretation of these data as indicating separate neural substrates comes from cortical blindness, where, following lesions to V1, patients have highly degraded vision in the contralateral field but some residual motion perception^46,47^. When viewing a reverse-phi version of the line motion illusion in his scotoma, patient GY showed preserved motion discrimination for phase, but not position cues^48^. It is thought that the residual motion vision in the scotoma may be mediated by the small proportion of parasol retinal ganglion cells that project to the superior colliculus^21^, with subsequent neurons projecting to cortical area V5/MT via the pulvinar nucleus^49^. Midget retinal ganglion cells are not thought to subserve the residual vision of patients with cortical blindness, as insufficient numbers project to the superior colliculus^21^, and there is complete transneuronal retrograde degeneration of the midget RGCs, but preservation of some parasol RGCs, following lesions of V1 in macaques^50^. Although there are still (inevitable) differences in the spatial scale of the phase and position cue in reverse-phi stimuli, where the phase cue is localised to the reversal of contrast at the edge and the position cue over the extent of the displacement, the preserved discrimination for phase but not position cues also supports our proposal that the parasol RGCs are the initial substrate for motion perceived from phase cues, and the midget RGCs for motion perceived from position cues.

Our investigation into the relationship of RGC topography and selective processing of phase and position cues for motion is purely correlational, between group level psychophysical HSFC with estimated parasol and midget RGCdistributions. There are large individual differences in the number of retinal ganglion cells between individuals^30^ which also vary with sex and age^51,52^, and which may extend to individual differences in the numbers of and relative topography of the midget and parasol RGCs. The vulnerability of midget and parasol retinal ganglion cells with age may differ, where evidence from neurodegenerative disorders shows an increased loss of parasol RGCs in patients with Alzheimer’s disease^53^ and glaucoma^54^, whereas in Parkinson’s disease^53^ and mitochondrial optic neuropathies^55^, there is greater loss of midget RGCs. In these patients, we might therefore expect to see a greater reliance on phase or position cues respectively, which could provide a more direct test of the involvement of different classes of retinal ganglion cells in processing different motion cues. Perhaps, with a better understanding of variations in thresholds and retinal ganglion cells densities between individuals, motion discrimination tests might also provide a means for early diagnoses of some of these pathological conditions. Furthermore, in macaques, there are differences in the overlap of the dendritic fields of the midget and parasol RGCs, at a ratio between 1.9 and 2.3 for the midget RGCs, and between 2 and 7 for the parasol RGCs^21^. If these factors vary between individuals, and especially if these vary non-uniformly across the retina, these could provide further individual differences between observers in the resolution of pathways subserved by midget and parasol retinal ganglion cells.

These factors could explain the differences between individuals in the relative decline in high spatial frequency cutoffs with eccentricity, represented in the differences in observers’ slope ratios. Nevertheless, despite the sources of variation, the correspondence between the averaged anatomical and psychophysical data represented by linear regressions is remarkably close: the slopes of the regression lines representing the linear ganglion cell densities fall clearly within the 95% confidence limits of the slopes of the corresponding psychophysical regressions.

Recent developments in imaging methods could be used to explore the impact of individual differences in anatomical data on psychophysical thresholds and provide a more direct interpretation of the role of parasol and midget RGCs in processing phase and position cues for motion. Models of cell density distributions for individual observers could be estimated from measurements taken by retinal imagining such as adaptive optics–optical coherence tomography (AO-OCT)^56^, (see review^57)^. Alternatively, a more direct test of this hypothesis could utilise fMRI techniques that can separately image the parvocellular and magnocellular layers of LGN (Denison et al*.*, 2014) and V1^58,59^. Imaging V1 has been used to demonstrate the impact of individual differences in cortical magnification on contrast sensitivity (Benson et al., 2021, Himmelberg et al., 2022), which could be of particular relevance to our hypothesis as cortical magnification varies between the midget and parasol RGCs^60,61,^^62^, and individual variation in cortical magnification factors could compound individual variations in ganglion cell topography. These imaging methods could be applied to this experiment, or indeed replications using other stimuli that disambiguate phase and position cues with a reduction or manipulation of the spatial scale covered by each cue, to investigate whether there is preferential activation of the respective parasol and midget layers of the LGN and V1 to phase and position cues.

In summary, we have provided evidence that motion direction discrimination thresholds based on phase cues and position cues are significantly different in peripheral retina, with thresholds for motion cued by position falling off more steeply than threshold for motion cued by phase. The relative change in thresholds with eccentricity for phase and position motion cues correlates with the relative change in linear cell density of parasol and midget ganglion retinal cells respectively. This suggests that the magnocellular (parasol) and parvocellular (midget) pathways could constitute separable neural substrates for first-order (Fourier) motion and third-order (feature-tracking) motion.

## Methods

### Method for Psychophysical experiment

#### Observers

Seven observers (three male) aged 21–57 years old, with normal or corrected-to-normal vision were included in this study. All observers gave written informed consent before participating in this study. This research received ethical approval from the Medical Sciences Inter-divisional Research Ethics Committee (IDREC) at the University of Oxford (R51336/RE001) and was conducted in accordance with the Declaration of Helsinki.

#### Apparatus

Stimuli were presented on a CRT monitor (Mitsubishi Diamond Pro 2070, 1600 × 1200 pixels), and gamma corrected using the ColorCAL MK II colorimeter (Cambridge Research Systems), with a maximum luminance of 81.33 cd/m^2^. Viewing distance was set at 180 cm from the monitor for stimuli viewed foveally and at 5° eccentricity, and at 110 cm for stimuli viewed at 10° and 15° eccentricity, and maintained with a chin rest. Viewing was monocular, with observers’ left eyes covered by an opaque eyepatch, stimuli were presented in the temporal visual field (*i.e.* onto the nasal hemiretina), and fixation was monitored using EyeLink 1000 eye tracker (S.R. Research, Ottowa, Canado.)

#### Stimuli

Two-frame apparent motion kinematograms were constructed from pairs of Gabor patches (sine-wave gratings weighted by a 2-dimensional Gaussian window) in MATLAB. The spatial frequency of the carrier gratings ranged from 0.3° to 32° with the standard deviation of the Gaussian window set to 2.5 cycles of the wavelength. The mean luminance of the Gabor patches was 40.67 cd/m^2^, with a Michelson’s contrast of 100%.

The range of spatial frequencies of the carrier in the Gabors presented at each of the four retinal locations (0°, 5°, 10° and 15° eccentricity) varied according to eccentricity to take into account variation in the contrast sensitivity function^45^. At the fovea, there were 7 different carrier spatial frequencies presented: 1, 2, 3.5, 6.1, 10.5, 18.4, and 32 cycles per degree. At 5° and 10° eccentricity, there were also 7 different carrier spatial frequencies presented: 0.5, 1, 2, 3.5, 6.1, 10.5 and 18.4 cycles per degree. At 15° eccentricity there were 6 different carrier spatial frequencies presented: 0.5, 1, 2, 3.5, 6.1 and 10.5 cycles per degree.

In any given trial, vertical (up or down) apparent motion was cued either by phase or by position: The phase cue was generated by shifting the phase of the sinusoidal carrier grating by a quarter cycle (90°) behind a static window between frames, while the position cue was generated by displacing the window by an equivalent distance while eliminating the phase cue by rotating the carrier 90° between frames. A third condition, in which apparent motion was cued simultaneously in opposite directions by phase and position was included in the tests, but the data are not presented in this paper, which focuses on assessing thresholds for the phase and position cues in isolation.

## Procedure

**Fig. 8:**
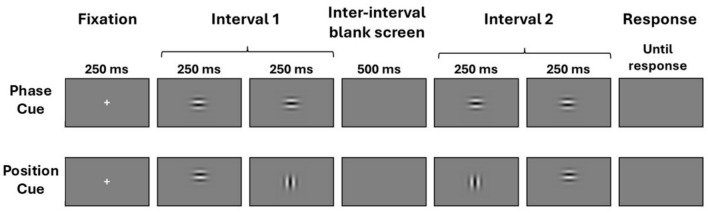
Top: The sequence of frames in a trial depicting apparent motion cued by phase (a 90° shift in the phase of the carrier grating while the Gaussian window remained stationary). In this case, upwards motion was depicted in interval 1. Bottom: The sequence of frames in a trial depicting apparent motion cued by position (displacement of the Gaussian envelope while the carrier grating was simultaneously rotated by 90° to eliminate any possible phase cue). In this case, upward motion was depicted in interval 2.

Observers were tested using a temporal, two-alternative forced choice (2AFC) procedure with vertical apparent motion, where each trial consisted of a Gabor where apparent motion was produced either by a change in the position of the gaussian window (‘position condition’), or a change in phase of the carrier of the sinusoidal carrier (‘phase condition’). Each trial contained two intervals separated by a blank interstimulus interval. Within each interval, motion was either in the upwards or downwards direction produced by an apparent motion change between frame 1 and frame 2. This procedure was the same for both phase and position cued apparent motion. In the second interval, following the blank interstimulus interval, the same pair of frames were presented in reverse order, to depict motion in the opposite direction. This produced trials consisting of 5 frames, with apparent motion of either upward motion followed by downward motion, or downward motion followed by upward motion (see Fig. 8). Observers were asked to report the interval in which they perceived upwards motion by keys ‘1’ and ‘2’ on a computer keyboard. The task was self-paced, with trials initiated by the observer pressing the spacebar.

Observers completed 45 trials per spatial frequency per condition, presented in a randomized order, producing 945 trials at foveal and 5° and 10° of eccentricity, and 810 trials when viewing stimuli at 15° of eccentricity. Each trial lasted 1250 ms with additional response time (250 ms fixation screen, 250 ms for each of the 4 stimulus frames, 500 ms inter-trial blank screen, and response interval). Observers completed the experiment over four sessions, each lasting between 90 and 120 min, with one eccentricity tested during each session, starting with foveal viewing and increasing in viewing eccentricity each session. Fixation was monitored using the Eyelink 1000 recording at 500 Hz, with an initial calibration session at the start of the experiment, and recalibrations during the experiment every 15 minutes. Trials that were aborted due to poor fixation were repeated, having been randomly inserted into the list of remaining trials.

### Threshold extraction

We calculated each observers’ high spatial frequency cutoff for the phase and position conditions at each of the 4 retinal locations tested. We plotted the mean proportion correct for the phase and position conditions at each eccentricity against log 10 of the spatial frequencies tested at each retinal location. These data were then fitted with a three-parameter quadratic function, separately for each condition and each eccentricity for each of the 7 observers. From these fitted functions we interpolated the spatial frequency corresponding to 75% proportion correct, which we refer to as the ‘high spatial frequency cutoff’ value, which corresponds to the phase or positional displacement threshold for direction discrimination.

### Models of ganglion cell density in the human retina

We reviewed the literature to find counts of retinal ganglion cells for human retinae. We used the model published by Drasdo (1989)^37^ based on 12 human retinae, and data for midget ganglion cells from Watson (2014)^13^, using data from 6 human retinae from Curcio & Allen (1990)^30^. We extrapolated estimates of parasol ganglion cell densities from Watson (2014)^13^ by taking the ratio between midget and parasol ganglion cells presented by Drasdo (1989)^37^ and applying this to Watson’s^13^ estimate of midget ganglion cells density. Both models have corrections applied to account for the foveal displacement of ganglion cells, where Drasdo (1989)^37^ uses the correction of Polyak & Klüver (1957)^63, and Watson (2014)^(^13uses the correction of Drasdo et al., (2007),^ and an additional correction for the asymmetry in distribution for ON and OFF mRGCs^64^^65^,(see Fig. 5 for final models).

### Comparisons between psychophysical thresholds and models of ganglion cell density

One method to compare observers’ high spatial frequency cutoffs for phase-cued and position-cued motion with the models of ganglion cell topography is to compare the rate of decline in sensitivity of each observer with the decrease in linear density with eccentricity of the parasol and midget retinal ganglion cells in the Watson (2014)^13^ and Drasdo (1989)^37^ models.

An additional method to compare between the cell density models and high spatial frequency cutoffs is to look at a possible similarity in the relative decline in density of the midget and parasol cells with eccentricity (for both for the Watson (2014)^13^ and Drasdo (1989)^37^ cell density data) and compare that to the relative decline in observers’ sensitivity to phase and position cues. This can be achieved by taking the ratio of the slopes for the fitted linear regressions to the log transformed data, using the steeper slope as the numerator:

$$\text{Psychophysical slope ratio}=\frac{{\text{Slope}}_{\text{position (observer)}}}{{\text{Slope}}_{\text{phase (observer)}}}$$

$$\text{Cell density slope ratio}=\frac{{\text{Slope}}_{\text{midget}}}{{\text{Slope}}_{\text{parasol}}}$$

These ratios of slopes describe the change in the relative number of midget and parasol ganglion cells with eccentricity. A slope ratio of 1 indicates no difference in the rate of decline in ganglion cell density with eccentricity between cell types. The calculated ratios of slopes indicate that midget ganglion cell density declines more steeply than parasol cell density with eccentricity, by 75.0% (ratio = 1.750^13^) or 78.7% (ratio = 1.787^37^).

## Data Availability

The datasets generated during and analysed during the current study are available from the corresponding author on reasonable request.
